# Accelerating digital health literacy for the treatment of growth disorders: The impact of a massive open online course

**DOI:** 10.3389/fpubh.2023.1043584

**Published:** 2023-04-18

**Authors:** Paul Dimitri, Luis Fernandez-Luque, Ekaterina Koledova, Shwetambara Malwade, Shabbir Syed-Abdul

**Affiliations:** ^1^NIHR Children and Young People MedTech Co-operative, Sheffield Children’s NHS Foundation Trust, Sheffield, United Kingdom; ^2^Adhera Health Inc., Palo Alto, CA, United States; ^3^Global Medical Affairs Cardiometabolic and Endocrinology, Merck KGaA, Darmstadt, Germany; ^4^Graduate Institute of Biomedical Informatics, Taipei Medical University, Taipei, Taiwan; ^5^School of Gerontology and Long-Term Care, Taipei Medical University, Taipei, Taiwan; ^6^International Center for Health Information Technology, Taipei Medical University, Taipei, Taiwan

**Keywords:** COVID-19, digital health, growth hormone deficiency, massive open online course, telemedicine

## Abstract

**Background:**

Growth hormone deficiency (GHD) is a rare disorder characterized by inadequate secretion of growth hormone (GH) from the anterior pituitary gland. One of the challenges in optimizing GH therapy is improving adherence. Using digital interventions may overcome barriers to optimum treatment delivery. Massive open online courses (MOOCs), first introduced in 2008, are courses made available over the internet without charge to a large number of people. Here, we describe a MOOC aiming to improve digital health literacy among healthcare professionals managing patients with GHD. Based on pre- and post-course assessments, we evaluate the improvement in participants’ knowledge upon completion of the MOOC.

**Methods:**

The MOOC entitled ‘Telemedicine: Tools to Support Growth Disorders in a Post-COVID Era’ was launched in 2021. It was designed to cover 4 weeks of online learning with an expected commitment of 2 h per week, and with two courses running per year. Learners’ knowledge was assessed using pre- and post-course surveys *via* the FutureLearn platform.

**Results:**

Out of 219 learners enrolled in the MOOC, 31 completed both the pre- and post-course assessments. Of the evaluated learners, 74% showed improved scores in the post-course assessment, resulting in a mean score increase of 21.3%. No learner achieved 100% in the pre-course assessment, compared with 12 learners (40%) who achieved 100% in the post-course assessment. The highest score increase comparing the pre- and the post-course assessments was 40%, observed in 16% of learners. There was a statistically significant improvement in post-course assessment scores from 58.1 ± 18.9% to 72.6 ± 22.4% reflecting an improvement of 14.5% (*p* < 0.0005) compared to the pre-course assessment.

**Conclusion:**

This “first-of-its-kind” MOOC can improve digital health literacy in the management of growth disorders. This is a crucial step toward improving the digital capability and confidence of healthcare providers and users, and to prepare them for the technological innovations in the field of growth disorders and growth hormone therapy, with the aim of improving patient care and experience. MOOCs provide an innovative, scalable and ubiquitous solution to train large numbers of healthcare professionals in limited resource settings.

## 1. Introduction

Growth hormone deficiency (GHD) is a rare disorder characterized by inadequate secretion of growth hormone (GH) from the anterior pituitary gland located in the fronto-central area of the brain (1). GHD can exist in isolation or as part of a multiple-pituitary hormone deficiency, given the role of the anterior pituitary gland in secreting other signaling hormones. GHD can be present from birth, as a result of abnormal pituitary gland development, or from genetic mutations leading to reduced hormone production or effect. It can also be acquired later in life as a result of trauma, infection, radiation therapy, or tumor growth within the brain. The majority of children diagnosed with GHD have no identifiable cause and are labeled as having “idiopathic” GHD. Globally, GHD has an estimated prevalence of one patient per 4,000–10,000 live births (2). GHD results in slow growth and short stature, and if left untreated, final height in adults will be significantly lower than their normal predicted adult height. Growth hormone signaling also affects body composition, metabolism, cardiovascular health, physical capacity, and bone strength, in addition to growth in the short and long term (3–5). Treatment is delivered through the daily administration of growth hormone injections under the skin (subcutaneous) from early childhood to adolescence. Achieving full height potential through recombinant human growth hormone (r-hGH) therapy relies on maintaining optimal dosing schedules and adherence to therapy (6–8). As a result, GHD leads to substantial emotional burden and can negatively affect patients’ quality of life (QoL) (2, 9–11). Up to 77% of patients treated with r-hGH are non-adherent to therapy as prescribed (12). Consequently, non-adherence adversely impacts therapeutic efficacy, often causing unnecessary dose increases, as well as increased healthcare utilization (13, 14).

One of the challenges in optimizing GH therapy is to improve adherence, particularly by addressing the barriers to optimum treatment delivery. Frequently reported concerns impacting patients’ QoL, and thus acting as a barrier to adherence, include injection discomfort, dissatisfaction with growth outcomes compared with pediatric endocrinologist predictions, lack of sophisticated dose individualization, challenges in clinic visits, and inadequate contact with healthcare professionals (HCPs). These barriers can be overcome through digital health interventions (15). Furthermore, appropriate patient engagement often calls for numerous face-to-face interactions, which can be challenging in clinical practice. In this regard, digital health solutions can offer more personalized support to patients and may ameliorate the need for regular contact with healthcare professionals, without the need to travel to consultations. The management of growth disorders is a complex and multifaceted area where response to therapy drives the outcome which is supported by adherence (16).

Individual patients have different needs, and these requirements vary by age and level of patient independence. This necessitates a “precision medicine” based approach to tailor therapy according to individual requirements. Digital tools already have the ability to support tracking of adherence to therapy and growth monitoring over time (17). New digital platforms can be developed to facilitate HCP-to-patient communication, deliver patient and HCP education, provide psychological support, and improve patient/caregiver engagement. Upcoming technological advances including virtual reality, augmented reality and gamification provide a means of better engagement, training, and support to patients, especially children (18). Gamification here refers to the persuasive system design by application of game principles and mechanics in healthcare environments. This encourages patients or users to increase user engagement thereby supporting adherence to therapy. This is intended to induce positive behavior by providing education in a format that the users understand and accept, making their experience fun-filled, bringing in mechanism to reduce anxiety associated with treatment. For achieving and sustaining optimal r-hGH therapy, it is incumbent on pediatric psychologists and HCPs to recognize, assess, and address the challenges to adherence through optimal patient-provider-caregiver communication, which again may be enhanced through digital tools to support monitoring and patient/caregiver feedback. Moreover, the data derived from the evolving, wider digital ecosystem has the potential to direct the development of new treatment paradigms and novel therapies. Future technological advances, coupled with continued educational support for HCPs, will further improve management of growth disorders and patient care while also driving healthcare policy development. Adopting an omnichannel approach, with digital touchpoints integrated with healthcare provision, may allow HCPs and patients to connect beyond therapy, enabling a patient-centric and data-driven experience thus underpinning a precision medicine approach to r-GH therapy. Competence and confidence in digital health literacy will be fundamental in the delivery of novel digital innovations that can be adopted by service users and providers.

Initially, there needs to be a recognition by service providers that digital tools add value to patient support and therapy. The COVID-19 pandemic accelerated healthcare adoption of technologies to deliver care, and in doing so this has led to a reliance on these technologies and the recognition that novel innovation can further improve healthcare and health education (19, 20). To promote this, there is a need to nurture HCPs with formal training that will improve digital literacy among caregivers and patients. The pandemic forced many to rethink and restructure the educational system, with a greater focus on distance learning. This learning medium was reported to be adaptable and rapid in delivery in different areas (21). According to a recent post-pandemic study on self-reported adaptability for postgraduate dental learners and instructors (in the context of abrupt transition to digital learning), both groups of stakeholders reported 81.15% adaptability (22). Massive open online courses (MOOCs), first introduced in 2008, are digital and online courses offering unlimited participation and open access *via* the internet as part of distance learning/education (23). Even before the onset of global pandemic in early 2020, MOOCs were growing rapidly and delivering high quality instructional content to the intended audiences. This multimodal eLearning model offers an innovative channel to equip large numbers of participants with digital health competencies. MOOCs present an opportunity to rapidly scale up digital health capabilities and act as a relevant source of knowledge for assessing patients’ needs, as previously demonstrated in diseases such as diabetes (24). With increasing awareness and acceptance of digital health technologies among both patients and HCPs, the need to integrate new digital tools into clinical care pathways to support service-users and service providers will accelerate. Previously, we have reported the development of a framework on the use of e-health solutions for the management of GHD (25). This framework maps directly onto the GHD patient pathway, opening digital health opportunities that are fully aligned with the need to improve digital health literacy through channels such as a MOOC. To our knowledge, there are no comparable examples available for the treatment of growth disorders. We present the method by which the MOOC, entitled “Telemedicine: Tools to Support Growth Disorders in a Post-COVID Era” was developed and launched in 2021. The MOOC was evaluated to determine the ability of this widely available educational digital platform in educating HCPs on digital health literacy in growth hormone therapy. Specifically, we present the curriculum and compare the scores for the pre-course and the post-course MOOC assessment responses among learners, and in addition present an evaluation of the learners’ comments.

## 2. Methods

### 2.1. Design and objectives of the MOOC

The MOOC entitled “Telemedicine: Tools to Support Growth Disorders in a Post-COVID Era” was launched on 6th September 2021 and designed to cover 4 weeks of online learning with an expected commitment of 2 h per week and with two courses running per year. This MOOC has unrestricted access and is available for all participants through FutureLearn platform *via* voluntary registration. The learners may receive access to courses of interest *via* a University/Institute subscription. Once registered, further information on related courses is automatically received.

The curriculum was conceptualized in collaboration and consensus between the authors, from Taipei Medical University Taiwan, The National Institute of Health Children and Young People MedTech Cooperative United Kingdom, and with educational sponsorship from Merck Healthcare KGaA, Darmstadt, Germany. Utilizing the eHealth Framework for GHD, key topics and skills required across the digital health patient journey were selected (26). These were then divided into different educational modules that were adapted to the MOOC multimodal eLearning model under the guidance of experts in medical education. Each week, participants had set objectives, access to online video content delivered by leaders in the field, interactive quizzes, and access to relevant literature or forums (26). The MOOC offered the opportunity to obtain Continuous Professional Development certification on completion.

### 2.2. Course participants

A total of 219 learners had enrolled on the MOOC by 17th December 2021. The enrolment was voluntary based on the interest of the learners. Informed consent from learners was collected while enrolling for the course. Learners who registered for the MOOC and attempted and completed all 10 questions in both the pre-course and post-course assessments were included in our analysis. The exclusion criteria included learners who only attempted either the pre- or the post-course assessment and learners who did not attempt all the questions in the pre-and post-course assessments. This study reports the learning performance of the learners, and the data was completely anonymous.

### 2.3. Content of the MOOC

The MOOC was designed as a guide on the use of digital interventions for HCPs and learners interested in digital health tools, using the management of growth disorders as a clinical example. The first week introduced eHealth in growth disorders, giving an overview of the eHealth Framework’s key concepts. The second week focused on research and evidence-based methods in eHealth. The third week was designed to explain methods for the involvement of patients and HCPs in the co-design of eHealth solutions, as a means to foster engagement and participation. Finally, the fourth week focused on communication between patients and their healthcare providers, including aspects related to mental wellbeing and technology-mediated communication. A detailed structure of the MOOC over the 4-week period is presented in Table 1. All comments posted by learners in the course discussion forum were considered in the evaluation.

**Table 1:** Content of the MOOC over 4 weeks.

Week	Content of MOOC		
Week 1	Challenges and opportunities in the diagnosis and management of growth disorders	What is digital health? Basic principles of digital health literacy	The application of digital health tools for the management of growth disorders now and in the future
Week 2	Methodological aspects of digital health research in chronic diseases	Principles of child health technology development—from proof of concept to commercialization	Using connected devices to develop real-world evidence on treatment adherence
Week 3	Working with patients and families: Unmet needs and co-design	Co-creation in digital health	How to use gamification for health behavioral change
Week 4	Technology-mediated communication between patients and healthcare providers	Introduction to behavioral psychology. Digital tools for supporting mental wellbeing for chronic conditions	Designing a personalized digital patient support program for patients and caregivers in growth disorders. Case study from TUITEK®

### 2.4. Web-based questionnaire

Learners’ knowledge was assessed using pre- and post-course surveys *via* the FutureLearn platform. The questions in these surveys were developed by educators specializing in their respective field. The questionnaire was structured as 10 multiple choice questions. For the full questionnaire please see the Supplementary material.

### 2.5. Statistical analysis

The *t*-test was used to determine the statistical significance in the percentage of pre-course and post-course scores of the learners. Significance was defined as *p* < 0.05. Data for pre- and post-course score assessment was analyzed anonymously.

## 3. Results

### 3.1. Pre- and post-course score assessment

**Figure 1:**
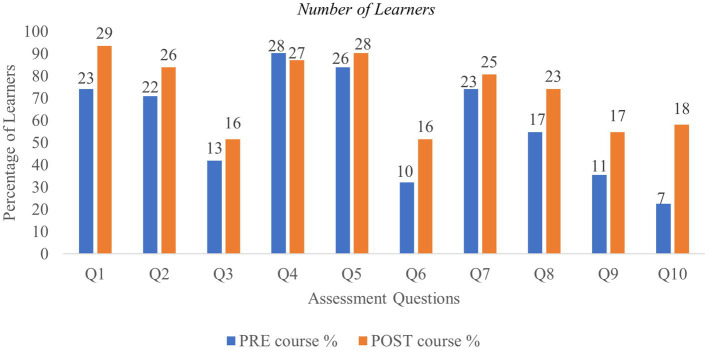
Correct responses (%) for individual questions in the pre- and post-course assessment.

**Figure 2:**
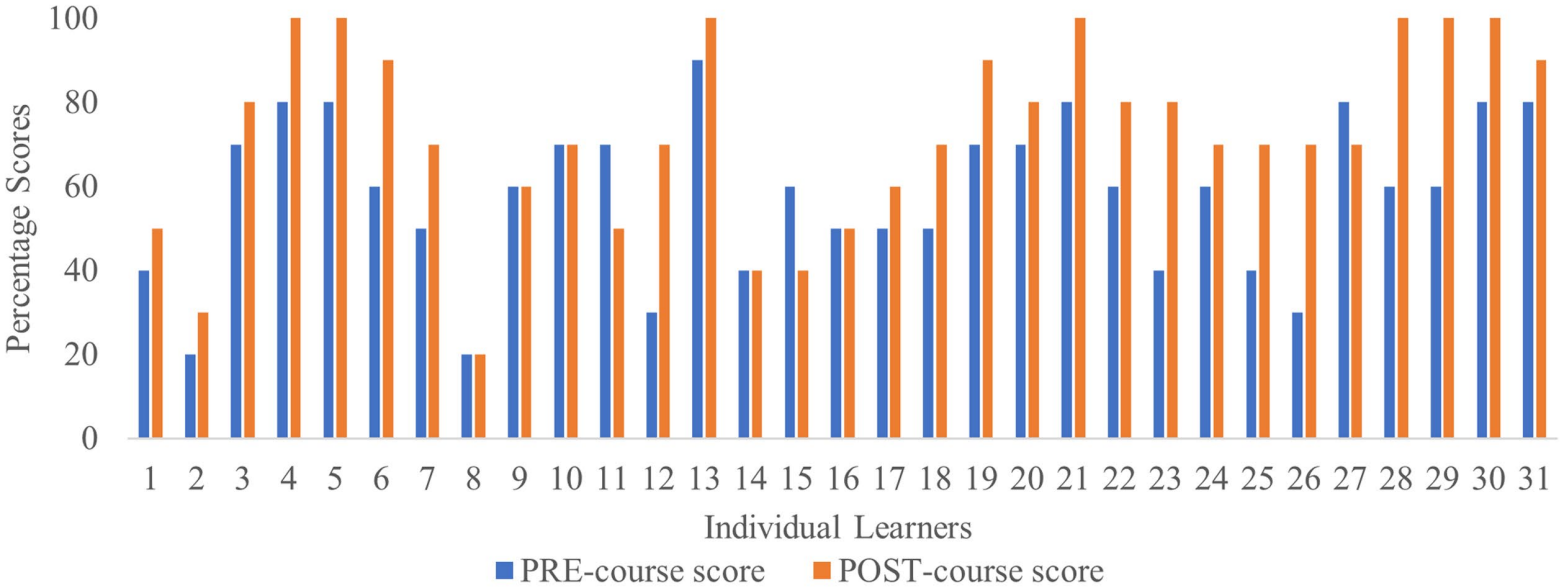
Score comparison in the pre- and post-course assessment for individual learners.

Thirty-one learners completed both the pre- and post-course assessments (Table 2). Of these, 74% showed improved scores in the post-course assessment, resulting in a mean score increase of 21.3% (Figure 1). Among the set of questions, Q3 and Q6 were perceived to be more difficult. Q3 dealt with virtual reality and digital platforms while Q6 was related to defining gamification in healthcare. Q4 related to the definition of medical informatics and was the only question where there was a slight reduction in the proportion of learners who answered correctly in the post course assessment, implying that understanding health informatics and technology may be challenging for some. Q10 was related to measurement instruments to assess QoL which is perceived to be a complex area. Pre- and post-course scores showed a significant change in the level of knowledge in this space after course completion (Figure 1). No learner achieved 100% in the pre-course assessment, while 12 learners (40%) achieved 100% in the post-course assessment. The highest score increase comparing the pre- and the post-course assessments was 40%, observed for 16% of learners (Figure 2). Learners 8, 9, 10, 14, and 16 obtained the same pre- and post-course scores, and 14 provided correct and incorrect answers for the same questions. Overall, an improvement in scores was observed for most learners in the post-course assessment. Only three learners (learners 11, 15 and 27) scored lower in the post-course assessment. Since demographics were not disclosed by learners, a detailed analysis could not be carried out to identify potential reasons for decreased scores (Figure 2). A statistically significant improvement in overall scores in the post-course assessment was observed as shown in Table 3, reflecting an improvement of 14.5% (*p* < 0.0005) compared to the pre-course assessment. The overall evaluation of the course assessments is listed in Table 4.

**Table 2:** Number of learners attempted and completed pre-and post-course assessment.

Number of learners	Pre-course assessment	Post-course assessment
Attempted	76	33
Completed	55	31

**Table 3:** T-test analysis for the statistical significance.

Variable	Pre-Mean (SD)	Post-Mean (SD)	*p*
Course percentage score	58.1 (18.9)	72.6 (22.4)	*p* < 0.0005

SD, standard deviation.

**Table 4:** Evaluation of pre-and post-course assessments.

Observation	Number
Number of learners who showed improvement in score in post-course assessment	23
Mean score increase among the 23 improved learners	21.8
Highest percentage score in the pre-course assessment	90%
Highest percentage score in the post-course assessment	100%
Number of learners who scored 100% in the post-course assessment	7
Highest percentage increase in score in post-course as compared to pre-course assessment	40%
Number of learners who showed 40% (highest) percentage increase in the scores	5
Number of learners who scored equally in pre- and post-course assessment	5
Number of learners who scored higher in pre-course as compared to post-course assessment	3
Least percentage score in the pre- and post-course assessment	20%

### 3.2. Evaluation of comments

Of the 132 comments analyzed between Week 1–4 (Table 5), 75 described an increase in knowledge, 39 stated that the course content was appropriate and 10 related to a positive impact on future learning and self-care (Table 6). Some learners commented on the potential applicability of the course content to other therapeutic areas.

**Table 5:** Evaluation of comments over Week 1–4.

	Week 1	Week 2	Week 3	Week 4
Evaluation of comments	Use of e-health in diagnosis and management of growth disorders	Research in e-health in the management of growth disorders	Strategies to foster engagement and collaboration to support the development of e-health tools	Patient to health care provider communication in the digital era Personalization behavioral support Future directions
Number of comments	48	19	29	36

**Table 6:** Description of the comments.

Topics	Number of comments	Example
Comments describing what learners learned for the particular step in every week. Learners added more information or description of information gained from the articles or the videos in the steps.	75	*“This was a very interesting article, which highlighted the advantages of the relatively recent development of many health apps, as well as the potential challenges (lack of health literacy) and risks (infringements on privacy for example)….”*
Comments giving positive feedback about the course/content being inspiring and insightful.	39	“*Thank you for all insights. It is very well designed course with lot of innovations and evidence based experiences shared by eminent speakers. Thank you all*.”
Comments including self-introduction of learners.	7	“Hi, I’m ** a retired neurologist from Italy interested in life-long learning, particularly in innovation in health care.”
Comments explaining their ongoing usage of telemedicine and wearable devices.	3	“*I’m using a continuous glucose monitoring system and a pedometer. Now I have a better control of my diabetes and I’m also reminded of walking at least 5,000 steps*”
General comments either curious to know more information or simple interactions among learners.	3	“This looks very interesting ‘Learner**’! Thank you for sharing.”
Comments irrelevant to the topic of the course.	5	*“La Conferencia de las Naciones Unidas sobre el Medio Ambiente y el Desarrollo, celebrada en Rio de Janeiro, Brasil, en junio de 1992 ….”*

## 4. Discussion

To our knowledge, this is the first MOOC developed to improve digital literacy in relation to the management of children with growth disorders. Our MOOC multimodal e-learning approach offers an innovative and scalable platform to train a large number of HCPs and other learners interested in digital health solutions for management of chronic diseases across different geographies. The digital health competencies addressed in our MOOC include the management of growth disorders through eHealth by HCP-patient engagement and patient empowerment, research and evidence-based methods in eHealth, and the use of e-health to support co-creation and co-design. The MOOC was developed to be of value to HCPs treating GHD and to also help HCPs and scientists to advance their entrepreneurial potential by providing insights into how eHealth solutions can be developed. Our analysis of learners’ capabilities prior to and after undertaking the MOOC has demonstrated that the MOOC can improve digital health literacy. Written feedback from learners demonstrated that the MOOC is capable of increasing knowledge, facilitated future learning and self-care, and that the course content developed was appropriate. Scores in four learners did not alter between pre- and post-course questionnaires and incorrect answers were given for the same questions by these learners at both stages. We speculate that this may be due to already well-established knowledge prior to undertaking the course and perceived lack of engagement to improve their knowledge in the relevant sections. For other MOOCs, typically around 5% of all enrolled learners attempted the assessment questionnaires (27, 28), whereas in our case 14% of the enrolled learners attempted and completed both the pre- and post-course assessments. The value of MOOCs has been previously reported, citing the educational benefits obtained by HCPs from developing countries who attended a 6-week online course in diabetes and obesity treatment therapies. More than 89% of the HCPs reported that the MOOC enhanced their professional skills and knowledge related to their future career. Importantly, MOOC-based distance learning can benefit the medical community by providing state-of-the-art, evidence-based guidelines, and readily available access to latest research, thus ultimately helping improve global health (24). The results from a MOOC on chronic pain, for example, demonstrated the impact of online courses in teaching innovative healthcare concepts and improving patients’ experience of care. The course democratized learning with a wider reach to diverse participants who can take such courses in any setting using an online computer, coupled with the self-paced nature of modules (29). Thus, MOOCs enhance educational outcomes as they provide direction to valid information (in comparison to online searches) and can improve knowledge and preferences related to treatment selection and use, as well as decrease health risks due to poor understanding of online information or its reliability.

Our group previously published a framework supporting the integration of digital health interventions into the patient journey to address unmet needs in treating pediatric growth disorders (25). Several workshops and questionnaires around eHealth tools and growth hormone therapy helped devise this e-health framework to support the identification of growth failure, diagnosis, implementation of therapy, and subsequent monitoring and support (25). Opportunities for better e-health support within this framework include offering enhanced patient/caregiver communication, medical and psychological support as children mature, optimized adherence, and increased access to educational opportunities for service users and providers. To ensure adoption and implementation of these digital health interventions, health centers need to be digitally prepared and ready to deploy novel virtual healthcare technologies (including online and offline electronic consulting), facilitated by the provision of e-learning educational resources (21). The MOOC was thus devised as a platform to address the need for improved educational access to support a “digital ready” workforce. Our MOOC adopted the same approach taken previously to develop the e-health framework, by considering digital health literacy and development in the context of the health journey undertaken by patients with growth disorders that require treatment with r-GH. As children with growth disorders need to have r-GH therapy for many years to achieve clinically significant outcomes, the development of a digital health ecosystem that connects patients, caregivers, and HCPs provides better opportunities for remote patient management, improved patient engagement, better adherence to therapy, and the adoption of a precision medicine approach tailored to the needs of the patient. To this end, our MOOC conveys knowledge and skills across different stakeholders to understand and enable the development of digital ecosystems to support long-term adherence to GH therapy in the context of the patient journey.

One of the limitations of our work is that only a small number of participants completed both the pre-and post-assessment questionnaires. Another limitation was the unavailability of learners’ demographic data to carry out analysis of scores and identify potential reasons for decreased scores of few learners post completion of MOOC. Nevertheless, our results still reflect a positive trend with a higher proportion of learners attempting both questionnaires compared with feedback for other MOOCs. In future, it will be important to assess whether learners have also gained skills and confidence in the use of digital health or showed any behavioral changes. It will also be important to make the course available in other languages. MOOCs provide a readily accessible and affordable way to improve digital health literacy to a large audience and are particularly crucial considering the rapid development of digital health underpinned by training for its safe and effective deployment.

## 5. Conclusion

Massive open online courses provide an innovative, scalable and ubiquitous solution to train large numbers of healthcare professionals in the limited resource settings. Our study demonstrates that the “Telemedicine: Tools to Support Growth Disorders in a Post-COVID Era” MOOC launched in 2021 is capable of improving digital health literacy in around three quarters of learners providing feedback. This is an important step toward improving the digital capability and confidence of healthcare providers and users in the field of growth disorders and growth hormone therapy with the aim of improving patient engagement and long-term care.

## Data availability statement

The datasets presented in this article are not readily available. Any requests for data by qualified scientific and medical researchers for legitimate research purposes will be subject to Merck’s Data Sharing Policy. Requests to access the datasets should be directed to https://www.merckgroup.com/en/research/our-approach-to-research-and-development/healthcare/clinical-trials/commitment-responsible-data-sharing.html.

## Ethics statement

Ethical review and approval was not required for the study on human participants in accordance with the local legislation and institutional requirements. The patients/participants provided their written informed consent to participate in this study.

## Author contributions

PD, LF-L, EK, SM, and SS-A made substantial contributions to the concept or design, acquisition, analysis and interpretation of data, and to the drafting of the manuscript or revising it critically for important intellectual content. All authors contributed to the article and approved the submitted version.

## Funding

This online course was made possible, thanks to an educational collaboration between Taipei Medical University and Merck Healthcare KGaA, Darmstadt, Germany. The course is designed for educational purposes and does not contain any kind of product promotion or marketing information. This work was supported by Merck (CrossRef Funder ID: 10.13039/100009945). This study received funding from Merck Healthcare KGaA, Darmstadt, Germany. The funder had the following involvement with the study: study design, data collection and analysis, decision to publish, and preparation of manuscript.

## Conflict of interest

EK is an employee of Merck KGaA, Darmstadt, Germany. LF-L is an employee at Adhera Health Inc., Palo Alto, CA, United States of America. PD has received consultancy fees for developing the MOOC.

The remaining authors declare that the research was conducted in the absence of any commercial or financial relationships that could be construed as a potential conflict of interest.

## Publisher’s note

All claims expressed in this article are solely those of the authors and do not necessarily represent those of their affiliated organizations, or those of the publisher, the editors and the reviewers. Any product that may be evaluated in this article, or claim that may be made by its manufacturer, is not guaranteed or endorsed by the publisher.
