# Supplementary material for: Accelerating digital health literacy for the treatment of growth disorders: The impact of a massive open online course
Source: Front Public Health. 2023 Apr 18;11:1043584. doi: 10.3389/fpubh.2023.1043584 (PMC10151751; doi:10.3389/fpubh.2023.1043584)
Supplement: Supplementary file 1 [file Data_Sheet_1.docx]

Supplementary Material

# Supplementary Material

**Pre-Post Assessment Questions**

1. What is eHealth according to the WHO?

***Options:***

1. The use of ICT (Information and Communication Technologies) for health.
2. The use of internet for health.
3. The use of Electronic Health Records.
4. None of these.

*Answer: A*

1. Which of the following e-health tools may improve the detection of growth disorders and management of patients on growth hormone?

***Options:***

1. Digital height measurement.
2. Digital health education.
3. Virtual reality distraction.
4. Online psychological support.
5. Data collection.
6. All of the above.

*Answer: F*

1. Which of these statements is true?
2. *Virtual reality can include a sense of touch.*
3. *Automated growth screening linked to an electronic patient record does not improve the identification of growth disorders.*
4. *Virtual reality cannot be used for pain distraction.*
5. *Digital platforms can be used to collect data on patient related outcome measures.*

***Options:***

- - 1. All of the above
    2. a, d
    3. a, b, c
    4. a, b, d

*Answer: B*

1. What is medical informatics research? Applied or basic science?

***Options:***

1. Medical informatics is always an applied discipline since it focuses on directly helping the medical practice.
2. Medical informatics can be both applied and basic science, since it might deal with the discovery of new basic biomedical knowledge (e.g. understanding genetics and health outcomes) and helping decision making in the clinical practice.
3. Both A and B.
4. None of these.

*Answer: B*

1. The following are important in Innovation Systems to integrate technology into healthcare:

***Options:***

1. Patient and family acceptance of the technology.
2. Cost of the technology.
3. Clinician Acceptance of the technology.
4. All of the above.

*Answer: D*

1. Gamification is the use of video games to increase engagement.

***Options:***

1. True.
2. False.
3. Cannot explain.
4. None of these.

*Answer: B*

1. The following are Principles of co-design:
2. Outcomes focused.
3. You must work in isolation
4. You must be respectful of others.
5. Co-design is adaptive.
6. Co-design can only be used by healthcare professionals.

***Options:***

- - 1. b, c, d
    2. a, c, d
    3. a, b, c, d

D. a, b, c, d, e

*Answer: B*

1. What are the most common uses of gamification in the health domain?

***Options:***

1. Support during clinical encounters and physical examinations since those are repetitive tasks that can be very boring.
2. There are only experiences in supporting patients with chronic pain.
3. Education and behavioral change since gamification can help to increase engagement and knowledge retention.
4. None of these.

*Answer: C*

1. The concept of “Health Related-Quality of Life” refers to:

***Options:***

1. A person's subjective perception of the impact of his/her health status on his/her functioning.
2. The severity of the symptoms of a disease and their impact on the person's physical health.
3. The clinician's judgement of a person's health status.
4. None of these.

*Answer: A*

1. When choosing a measurement instrument to assess the quality of life in a child/adolescent, one should consider the following:

***Options:***

1. Choose the instrument that is the best psychometrically (reliable and valid), regardless of the specific population to which it is to be applied.
2. Choosing an instrument that specifically fits the needs of our context and target population.
3. Choose an instrument that comprehensively assesses all components of quality of life and is generic to all medical conditions, and ages.
4. None of these.

*Answer: B*
